# Clinical Features and Genomic Characterization of Post-Colonoscopy Colorectal Cancer

**DOI:** 10.14309/ctg.0000000000000246

**Published:** 2020-10-06

**Authors:** Hidenori Tanaka, Yuji Urabe, Shiro Oka, Yasutsugu Shimohara, Tomoyuki Nishimura, Katsuaki Inagaki, Yuki Okamoto, Kenta Matsumoto, Ken Yamashita, Kyoku Sumimoto, Yuki Ninomiya, Ryo Yuge, Shinji Tanaka, Kazuaki Chayama

**Affiliations:** 1 Department of Gastroenterology and Metabolism, Hiroshima University Hospital, Hiroshima, Japan;; 2 Division of Regeneration and Medicine Center for Translational and Clinical Research, Hiroshima University Hospital, Hiroshima, Japan;; 3 Department of Endoscopy, Hiroshima University Hospital, Hiroshima, Japan.

## Abstract

Some colorectal cancers (CRCs) may be missed during colonoscopies. We aimed to determine the clinicopathological, biological, and genomic characteristics of post-colonoscopy CRCs (PCCRCs).

**METHODS:**

Of the 1,619 consecutive patients with 1,765 CRCs detected between 2008 and 2016, 63 patients with 67 PCCRCs, when colonoscopies were performed 6–60 months before diagnosis, were recruited. After excluding patients with inflammatory bowel disease, familial polyposis syndrome, CRCs that developed from diminutive adenomatous polyps, and recurrent CRCs after endoscopic resection, 32 patients with 34 PCCRCs were enrolled. The lesions' clinicopathological features, mismatch repair proteins (MMRs), and genomic alterations were investigated.

**RESULTS:**

The overall PCCRC-5y rate, rate of intramucosal (Tis) lesions, and rate of T1 or more deeply invasive cancers were 3.7% (66/1,764), 3.9% (32/820), and 3.6% (34/944), respectively. Thirty-three patients' MMRs were investigated; 7 (21%) exhibited deficient MMRs (dMMRs), comprising 4 with T2 or more deeply invasive cancers and 5 whose lesions were in the proximal colon. Twenty-three tumors' genomic mutations were investigated; *PIK3CA* had mutated in 5 of 6 T2 or more deeply invasive cancers, of which, 4 were located in the proximal colon. Two patients with dMMRs and *BRAF*^*V600E*^ mutations had poor prognoses. Sixty-one percent (17/28) of the macroscopic type 0 lesions were superficial. All superficial Tis and T1 PCCRCs were detected <24 months after the negative colonoscopies. They were distributed throughout the colon and rectum.

**DISCUSSION:**

PCCRCs may be invasive cancers in the proximal colon that exhibit dMMRs and/or *PIK3CA* mutations or missed early CRCs especially superficial lesions.

## INTRODUCTION

Although the incidence of and mortality associated with colorectal cancer (CRC) are reduced with colonoscopy (1–11), some CRCs, namely post-colonoscopy CRCs (PCCRCs), are diagnosed months or years after negative colonoscopies. PCCRCs are missed at previous colonoscopies (12–17) because of poor bowel preparation and no cecal intubation (13–15), due to postendoscopic resection (ER) lesional recurrences (13,16,17), or lesions with rapid growth potentials (9). PCCRCs are associated with female sex (14,18–21), proximal colon cancer (12,14,16–18,20–29), older age (12,18,20–23,27), increased comorbidity (18–20,24), and high microsatellite instability (9,23,26–30). PCCRC prognosis varies widely; it can be better than (25), the same as (16,20,26,28,29), or worse than (18) those of other CRCs. However, intramucosal (Tis) cancers, as defined by the Japanese (31) and *TNM Classification of Malignant Tumors* (32) criteria, were not included in these studies. Evaluating Tis lesions determines the progress and prognoses of patients with PCCRCs because they are precursors of invasive cancers (33,34). Moreover, no studies have investigated the genomic characteristics of PCCRCs, except some gene mutations, including *KRAS* and *BRAF* (9,23,27,29,30). Although most PCCRCs are considered lesions that were missed at previous colonoscopies (13,20), they may have biological features different from those of other CRCs. This study aimed to determine the clinicopathological, biological, and genomic characteristics of PCCRCs.

## METHODS

### Patient enrollment

This retrospective, single-center study recruited 1,619 consecutive patients with 1,765 CRCs diagnosed at Hiroshima University Hospital between January 2008 and December 2016. The colonoscopy dates and results were retrieved from electronic medical records and colonoscopy reports. Patients who had ≥1 negative colonoscopies 6–60 months before a CRC diagnosis were extracted as patients with PCCRC. To investigate the characteristics of PCCRCs, which had an undetermined etiology, and evaluate the reason for them having been missed during the colonoscopies, PCCRCs with definitive causes or obvious carcinogenic factors were excluded. Therefore, the exclusion criteria were as follows: inflammatory bowel disease, familial polyposis syndrome, CRCs developed from previously detected diminutive adenomatous polyps, and recurrent CRC after ER. The study was performed in accordance with the Declaration of Helsinki, and its protocol was approved by Hiroshima University's Institutional Review Board (E-1371).

### Definitions

“Negative colonoscopy” was defined as a colonoscopy that was negative for CRC or a colonoscopy wherein all CRCs had undergone ER. PCCRC was defined as a CRC with a history of ≥1 negative colonoscopies performed 6–60 months before its diagnosis to minimize contamination with diagnostic examinations (23). CRC with a history of colonoscopy performed <6 or >60 months before diagnosis was defined as a “detected CRC.” “Multiple negative colonoscopies” refer to patients with histories of ≥1 negative colonoscopies performed <60 months before the last negative colonoscopy. The PCCRC rate was calculated as the number of PCCRCs (including cases of inflammatory bowel disease, familial polyposis syndrome, CRCs developed from previously detected diminutive adenomatous polyps, and recurrent CRC after ER)/the total number of CRCs (PCCRCs + detected CRCs) × 100 (18), which was described as the PCCRC-5y rate. The PCCRC rate with a colonoscopy interval of 6–36 months was also evaluated, and this was described as the PCCRC-3y rate (35).

All colonoscopies were performed by endoscopic experts with experience performing >1,000 total colonoscopies. A Tis lesion was defined as intramucosal cancer according to the Japanese Society for Cancer of the Colon and Rectum's (31) and the *TNM Classification of Malignant Tumors* (32) criteria, which correspond with high-grade dysplasia in the World Health Organization classification (36).

### Evaluation of clinicopathological characteristics

Data were collected from medical records, electronically stored colonoscopic images and reports, and histology reports that described the patient's age and sex; treatment options; death from cancer; history of adenoma; synchronous/metachronous CRCs; family history of CRC; time to a CRC diagnosis from the previous colonoscopy; history of multiple negative colonoscopies; the cancer location, namely, the proximal colon, including the cecum and ascending/transverse colon, distal colon, including the descending/sigmoid colon, and rectum; the lesion's macroscopic classification; histologic types; T stage; TNM staging; and lymph node or other organ metastases.

### Immunohistochemistry

Mismatch repair proteins (MMRs), including MLH1, MSH2, MSH6, and PMS2, were analyzed immunohistochemically. Formalin-fixed and paraffin-embedded (FFPE) sections were deparaffinized and rehydrated. Heat-induced antigens were retrieved using a microwave and citrate buffer. After blocking endogenous peroxidase and nonspecific protein binding, the sections were incubated with antibodies to MLH1 (BD Pharmingen, Franklin Lakes, NJ), MSH2 (Bio-Genex Laboratories, Fremont, CA), MSH6 (Bio-Genex Laboratories), and PMS2 (Bio-Genex Laboratories), followed by incubation with a Dako REAL EnVision Detection System (Peroxidase/DAB^+^, Rabbit/Mouse Code K5007; Agilent Technologies, Santa Clara, CA). The antigen-antibody reaction was visualized using diaminobenzidine, and the sections were counterstained with hematoxylin. The tumor's proteins were classified as MMR-deficient (dMMR) or MMR-proficient (pMMR).

### Tissue capture and DNA extraction

Tumors and normal colorectal mucosa were visually dissected from several 10-μm FFPE sections. The DNA was extracted using the GeneRead DNA FFPE kit (Qiagen GmbH, Hilden, Germany), eluted into buffer (40 μL), and quantified using the Qubit dsDNA HS kit (Thermo Fisher Scientific, Waltham, MA) and a Qubit 1.0 fluorometer (Thermo Fisher Scientific). The quantities and quality of the FFPE-derived DNA were ascertained by determining the normalized DNA integrity score obtained from the quantitative polymerase chain reaction using an NGS FFPE QC Kit (Agilent Technologies).

### Target enrichment and next-generation sequencing

DNA from the tumors and normal colorectal mucosa was fragmented into 150–200 base pairs using a restriction enzyme and the SureSelect XT HS Kit (Agilent Technologies) and the XT Low Input Enzymatic Fragmentation Kit (Agilent Technologies); the fragments were used to construct libraries according to the manufacturer's instructions. The DNA (≥109 ng) (range 109–2,910 ng) was prepared for sequencing. The exons of 90 oncogenes and the associated introns of 35 fusion oncogenes were enriched using the SureSelect-XT HS NCC oncopanel (Agilent Technologies) (see Table, Supplementary Digital Content 1, http://links.lww.com/CTG/A401). The pooled libraries underwent quality control checks using the High Sensitivity D1000 ScreenTape Assay (Agilent Technologies) and the 2200 TapeStation System (Agilent Technologies). Sequencing was performed with paired-end reads using the HiSeq X platform (Illumina, San Diego, CA).

### Variant detection

The sequencing reads were aligned to the hg19 reference sequence and analyzed using SureCall Software, version 4.1 (Agilent Technologies). To improve mapping quality before variant calling, polymerase chain reaction duplicates were removed using molecular barcodes and SureCall Software, version 4.1. Paired-end and single-end analyses using SureCall Software, version 4.1, were used to identify single nucleotide variants and insertions/deletions (indels) in the tumors. Called variants in the normal colorectal mucosa were also considered germline mutations. To reduce the false-positive rate, we set cutoff values for somatic mutations in tumors, as follows: read depth >20 and forward/reverse balance between 0.25 and 0.75. We configured the SureCall SNP caller using the SureSelect default settings, as follows: variant score threshold: 0.3, minimum quality for base: 30, variant call quality threshold: 100, minimum allele frequency: 0.1, and minimum number of reads supporting the variant allele: 10. Variants that were repeat sequences registered in the RepeatMasker program (University of California Santa Cruz, Santa Cruz, CA) and called as replacements or clearly identified as sequencing errors in the Integrative Genomic Viewer (Broad Institute of Massachusetts Institute of Technology and Harvard, Cambridge, MA) were excluded from all samples as somatic mutation candidates. The somatic mutations were classified as follows: category I: frameshift indels or nonsense mutations; category II: missense mutations; and others, including synonymous changes, mutations located at introns, or intergene mutations (see Tables, Supplemental Digital Content 2 and 3, http://links.lww.com/CTG/A402 and http://links.lww.com/CTG/A403).

## RESULTS

**Figure 1.**
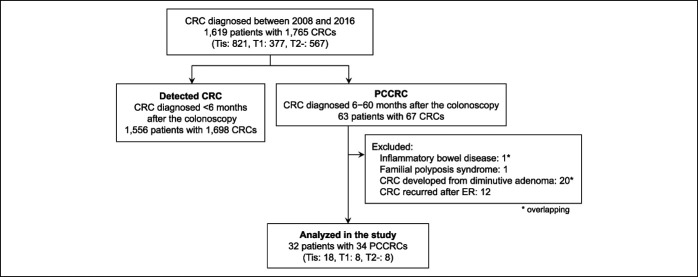
Flowchart of the enrolled patients. Thirty-two patients with 34 PCCRCs, including 18 intramucosal, 8 T1, and 8 T2 or more deeply invasive cancers, were eligible for this study. ER, endoscopic resection; PCCRC, post-colonoscopy colorectal cancer.

Of the 1,619 patients with 1,765 CRCs, 63 patients with 67 CRCs were classified as having PCCRCs. Of the 67 PCCRCs, 33, 18, and 16 were Tis, T1, and T2 or more deeply invasive cancers, respectively. One patient had 2 synchronous Tis PCCRCs. The overall PCCRC-5y rate was 3.7% (66/1,764). The rate of Tis, T1, and T2 or more deeply invasive cancers was 3.9% (32/820), 4.8% (18/377), and 2.8% (16/567), respectively (Table 1). After excluding patients who met the exclusion criteria, 32 patients with 34 PCCRCs were analyzed in the study (Figure 1). Of the 34 eligible PCCRCs, 18, 8, and 8 were Tis, T1, and T2 or more deeply invasive cancers, respectively.

**Table 1.**
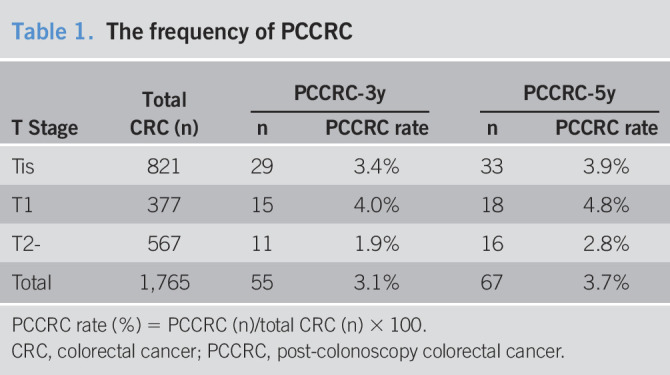
The frequency of PCCRC

T Stage	Total CRC (n)	PCCRC-3y		PCCRC-5y	
		n	PCCRC rate	n	PCCRC rate
Tis	821	29	3.4%	33	3.9%
T1	377	15	4.0%	18	4.8%
T2-	567	11	1.9%	16	2.8%
Total	1,765	55	3.1%	67	3.7%

PCCRC rate (%) = PCCRC (n)/total CRC (n) × 100.

CRC, colorectal cancer; PCCRC, post-colonoscopy colorectal cancer.

### Clinicopathological features of PCCRCs

Table 2 shows the clinicopathological features of the PCCRCs. More men (79%) than women (21%) had PCCRCs. Approximately 50% of PCCRCs were located in the proximal colon. Of the macroscopic type 0 PCCRCs, 61% (17/28) were superficial lesions. Most patients had early cancer, and all the PCCRCs were resectable with ER alone, additional surgery after ER, or initial surgery. Eight PCCRCs were metachronous cancers, 4 of which had a history of a colectomy, and 3 had histories of ER at a different site of the colorectum.

**Table 2.**
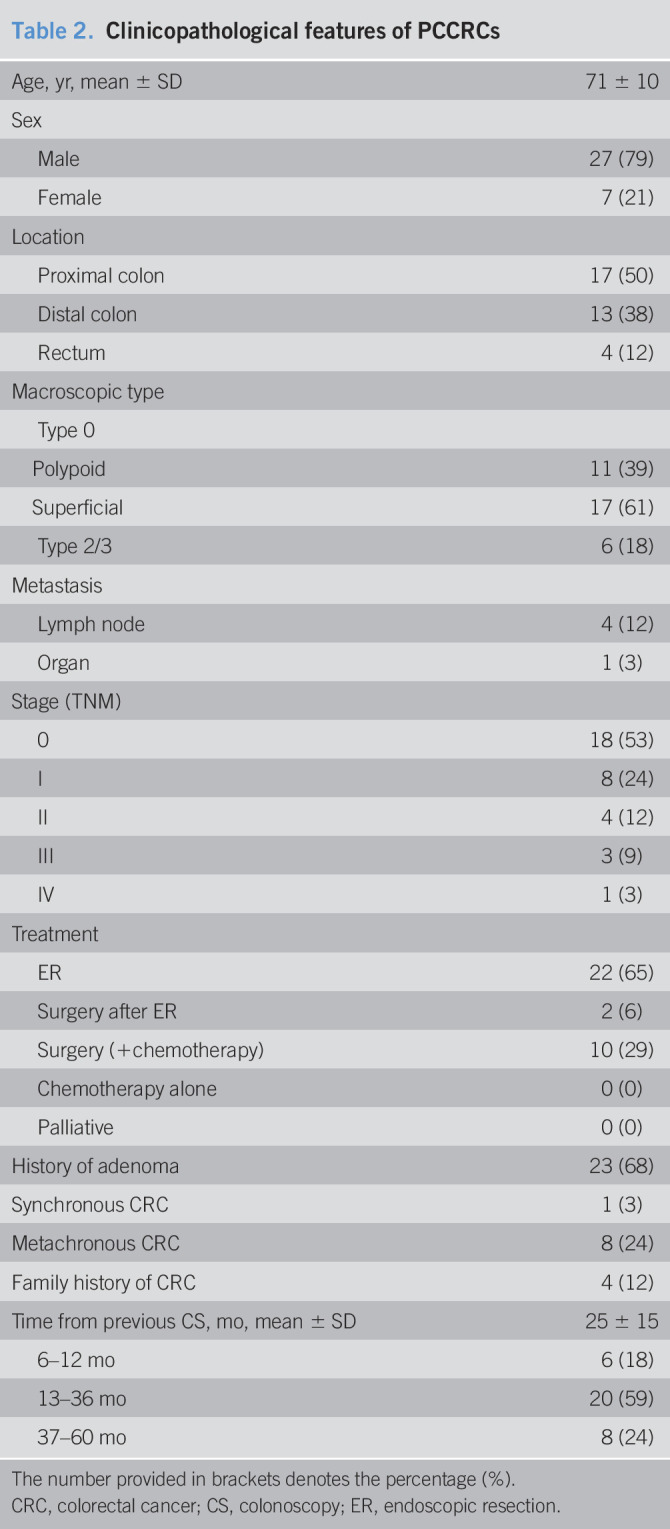
Clinicopathological features of PCCRCs

Age, yr, mean ± SD	71 ± 10
Sex	
Male	27 (79)
Female	7 (21)
Location	
Proximal colon	17 (50)
Distal colon	13 (38)
Rectum	4 (12)
Macroscopic type	
Type 0	
Polypoid	11 (39)
Superficial	17 (61)
Type 2/3	6 (18)
Metastasis	
Lymph node	4 (12)
Organ	1 (3)
Stage (TNM)	
0	18 (53)
I	8 (24)
II	4 (12)
III	3 (9)
IV	1 (3)
Treatment	
ER	22 (65)
Surgery after ER	2 (6)
Surgery (+chemotherapy)	10 (29)
Chemotherapy alone	0 (0)
Palliative	0 (0)
History of adenoma	23 (68)
Synchronous CRC	1 (3)
Metachronous CRC	8 (24)
Family history of CRC	4 (12)
Time from previous CS, mo, mean ± SD	25 ± 15
6–12 mo	6 (18)
13–36 mo	20 (59)
37–60 mo	8 (24)

The number provided in brackets denotes the percentage (%).

CRC, colorectal cancer; CS, colonoscopy; ER, endoscopic resection.

Table 3 shows the clinicopathological features of the PCCRC according to the T stage. T2 or more deeply invasive cancers were absent in the rectum and were more likely to be in the proximal colon than Tis and T1 cancers. All the T2 or more deeply invasive cancers were diagnosed >12 months after the negative colonoscopies, while 3 of 18 Tis and 3 of 8 T1 cancers were diagnosed within 12 months of previous negative colonoscopies. Sixty-two percent (21/34) of patients had a history of multiple negative colonoscopies. Cecal intubation was achieved in all negative colonoscopies; however, poor bowel preparation was observed in 2 of 18 negative colonoscopies performed in patients with Tis cancers. The 3-year survival rates, determined using the Kaplan-Meier method, for patients with Tis, T1, and T2 or more deeply invasive cancers were 94%, 75%, and 60%, respectively; 2 patients who died from T2 or more deeply invasive cancers had poorly differentiated or mucinous adenocarcinomas with peritoneal disseminations.

**Table 3.**
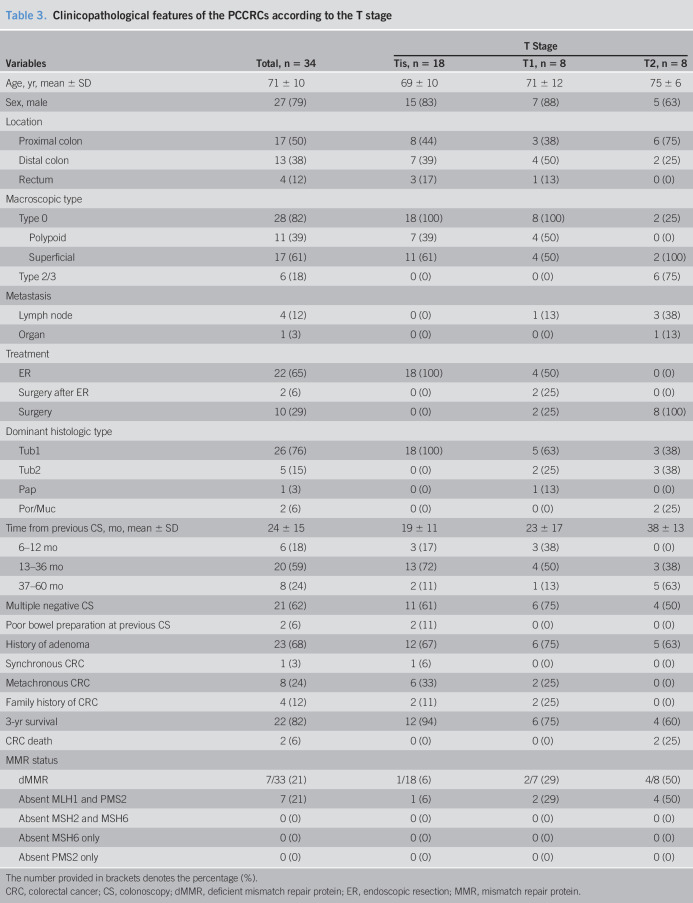
Clinicopathological features of the PCCRCs according to the T stage

Variables	Total, n = 34	T Stage		
		Tis, n = 18	T1, n = 8	T2, n = 8
Age, yr, mean ± SD	71 ± 10	69 ± 10	71 ± 12	75 ± 6
Sex, male	27 (79)	15 (83)	7 (88)	5 (63)
Location				
Proximal colon	17 (50)	8 (44)	3 (38)	6 (75)
Distal colon	13 (38)	7 (39)	4 (50)	2 (25)
Rectum	4 (12)	3 (17)	1 (13)	0 (0)
Macroscopic type				
Type 0	28 (82)	18 (100)	8 (100)	2 (25)
Polypoid	11 (39)	7 (39)	4 (50)	0 (0)
Superficial	17 (61)	11 (61)	4 (50)	2 (100)
Type 2/3	6 (18)	0 (0)	0 (0)	6 (75)
Metastasis				
Lymph node	4 (12)	0 (0)	1 (13)	3 (38)
Organ	1 (3)	0 (0)	0 (0)	1 (13)
Treatment				
ER	22 (65)	18 (100)	4 (50)	0 (0)
Surgery after ER	2 (6)	0 (0)	2 (25)	0 (0)
Surgery	10 (29)	0 (0)	2 (25)	8 (100)
Dominant histologic type				
Tub1	26 (76)	18 (100)	5 (63)	3 (38)
Tub2	5 (15)	0 (0)	2 (25)	3 (38)
Pap	1 (3)	0 (0)	1 (13)	0 (0)
Por/Muc	2 (6)	0 (0)	0 (0)	2 (25)
Time from previous CS, mo, mean ± SD	24 ± 15	19 ± 11	23 ± 17	38 ± 13
6–12 mo	6 (18)	3 (17)	3 (38)	0 (0)
13–36 mo	20 (59)	13 (72)	4 (50)	3 (38)
37–60 mo	8 (24)	2 (11)	1 (13)	5 (63)
Multiple negative CS	21 (62)	11 (61)	6 (75)	4 (50)
Poor bowel preparation at previous CS	2 (6)	2 (11)	0 (0)	0 (0)
History of adenoma	23 (68)	12 (67)	6 (75)	5 (63)
Synchronous CRC	1 (3)	1 (6)	0 (0)	0 (0)
Metachronous CRC	8 (24)	6 (33)	2 (25)	0 (0)
Family history of CRC	4 (12)	2 (11)	2 (25)	0 (0)
3-yr survival	22 (82)	12 (94)	6 (75)	4 (60)
CRC death	2 (6)	0 (0)	0 (0)	2 (25)
MMR status				
dMMR	7/33 (21)	1/18 (6)	2/7 (29)	4/8 (50)
Absent MLH1 and PMS2	7 (21)	1 (6)	2 (29)	4 (50)
Absent MSH2 and MSH6	0 (0)	0 (0)	0 (0)	0 (0)
Absent MSH6 only	0 (0)	0 (0)	0 (0)	0 (0)
Absent PMS2 only	0 (0)	0 (0)	0 (0)	0 (0)

The number provided in brackets denotes the percentage (%).

CRC, colorectal cancer; CS, colonoscopy; dMMR, deficient mismatch repair protein; ER, endoscopic resection; MMR, mismatch repair protein.

### Biological characteristics

Thirty-three tumors were analyzed immunohistochemically for MLH1, MSH2, MSH6, and PMS2; one T1 tumor was excluded because an FFPE block containing the tumor was unavailable. Seven PCCRCs were dMMRs, all of which showed absent MLH1 and PMS2 (Table 3). dMMRs were observed in 50%, 29%, and 6% of the T2 or more deeply invasive, T1, and Tis cancers, respectively.

**Figure 2.**
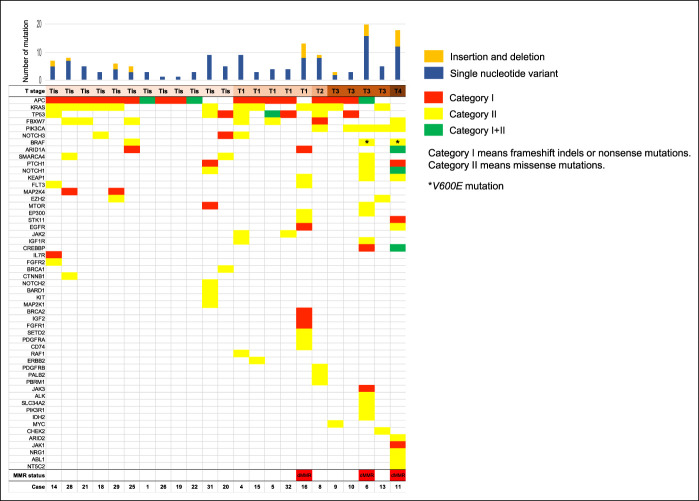
Mutational landscapes in the 23 PCCRCs. The upper panel shows the number of somatic mutations per sample; the blue bars indicate the number of single nucleotide variants, and the orange bars indicate the number of insertions and deletions. The lower panel shows the types of genomic mutations, T stage, and the dMMR status. The red, yellow, and green cells represent genes with category I mutations (frameshift indels or nonsense mutations), category II mutations (missense mutations), and category I and II mutations, respectively. The asterisks represent the *BRAF*^*V600E*^ mutations. dMMR, deficient mismatch repair protein; PCCRC, post-colonoscopy colorectal cancer.

We purified DNA from 33 paired PCCRCs and normal tissues, but 10 patients' DNAs were of insufficient quality and/or quantity for sequencing. Therefore, we analyzed 23 PCCRCs with DNA of sufficient quality. Figure 2 shows the mutation landscapes of 23 PCCRCs. *BRAF* and *KRAS* were mutated in 3 (13%) and 12 (52%) cases, respectively. Of the 3 cases with *BRAF* mutations, 2 had missense *BRAF*^*V600E*^ mutations coexisting with dMMRs. No cases of Tis or T1 PCCRCs had *PIK3CA* mutations, and 5 of 6 T2 or more deeply invasive PCCRCs had *PIK3CA* missense mutations. Next-generation sequencing of DNA from 3 cases with dMMRs showed hypermutations with large numbers of somatic mutations (tumor mutational burden >10). A patient had suspected Lynch syndrome (case 16): One had 2 first-degree relatives with CRC, and developed CRC before 50 years, which had dMMRs but not the *BRAF* mutation; their MMR gene germline mutations were not investigated.

### Relationship among the colonoscopy intervals and the clinical and biological characteristics

**Figure 3.**
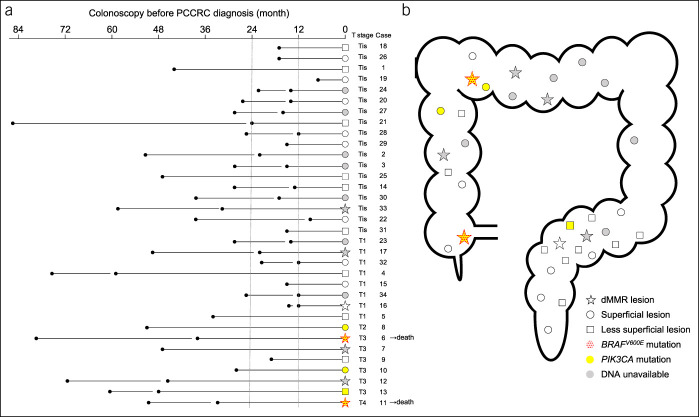
Relationship between the colonoscopy intervals, T stages, superficial lesions, MMR immunohistochemistry, and *BRAF/PIK3CA* mutations. (**a**) Colonoscopy intervals for each case. (**b**) The distribution of the PCCRCs. The circles and squares represent the superficial and less superficial lesions, respectively. The stars indicate tumors with dMMRs; every lesion with dMMR is not superficial. The dotted red and filled yellow represent tumors with *BRAF*^*V600E*^ and *PIK3CA* mutations, respectively. Both of the tumors with *BRAF*^*V600E*^ mutations have *PIK3CA* mutations. The filled gray indicates tumors for which DNA is unavailable. dMMR, deficient mismatch repair protein; MMR, mismatch repair protein; PCCRC, post-colonoscopy colorectal cancer.

**Figure 4.**
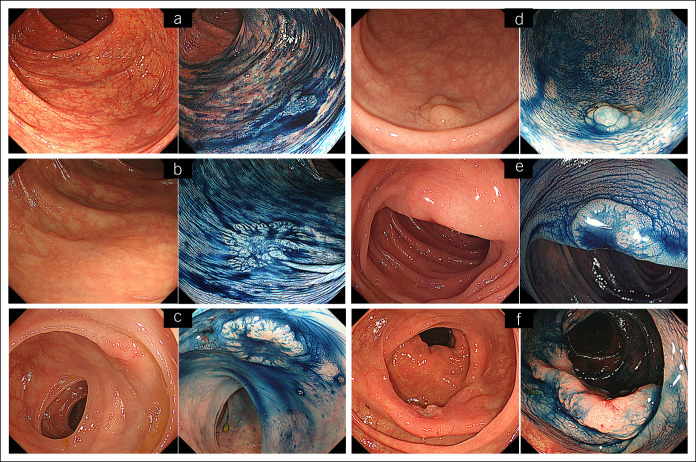
Representative images of superficial PCCRCs. The white light images and their corresponding chromoendoscopy images are shown for each case. (**a**) Case 2: Tis tumor in the ascending colon. (**b**) Case 3: Tis tumor in the transverse colon. (**c**) Case 19: Tis tumor in the sigmoid colon. (**d**) Case 28: Tis tumor in the upper rectum. (**e**) Case 32: T1 tumor in the sigmoid colon. (**f**) Case 10: T3 tumor in the ascending colon. PCCRC, post-colonoscopy colorectal cancer.

Figure 3a shows the intervals between the PCCRC diagnoses and previous colonoscopies in the context of the T stage, tumor morphology, and MMR immunohistochemistry in all cases. The intervals between the previous negative colonoscopies and the diagnoses of superficial Tis and T1 PCCRCs were <24 months and mostly around 12 months. None of the superficial lesions exhibited dMMRs. Compared with the PCCRCs exhibiting pMMRs, those exhibiting dMMRs tended to be detected later when they had invaded more deeply. One T3 PCCRC detected within 24 months of a negative colonoscopy exhibited pMMRs (case 9); this was the only tumor with an *MYC* missense mutation. Figure 3b shows the distribution of the PCCRCs within the colon and rectum. Cancers exhibiting dMMRs were more likely to be located in the proximal colon. Superficial lesions were distributed evenly throughout the entire colon and rectum. Less superficial lesions, including type 2/3 lesions and type 0 lesions with polypoid morphologies, were more likely to be located in the sigmoid colon. Figure 4 presents endoscopic images of superficial PCCRCs. Many of the Tis and T1 cancers had superficial appearances and coloring that was similar to that of the surrounding normal mucosa and were considered difficult to detect. Both the PCCRCs with dMMRs and *BRAF*^*V600E*^ mutations were located in the proximal colon, had peritoneal disseminations, and caused the patients' deaths (see Figure, Supplemental Digital Content 4, http://links.lww.com/CTG/A404).

## DISCUSSION

This study's findings revealed the clinicopathological and biological characteristics of PCCRCs. Although most PCCRCs are considered lesions that were missed at previous colonoscopies (13,20), this is difficult to prove scientifically; a missed lesion may be a rapidly developing cancer. To investigate this issue, we included Tis lesions, excluded CRCs that had recurred after ER, and examined the MMR status and genomic landscapes of PCCRCs as well as the relationship with morphology and location.

In this study, the PCCRC-3y and PCCRC-5y rates for T1 or more deeply invasive cancers were 2.8% (26/944) and 3.6% (34/944), respectively, which were relatively low compared with previously reported rates of 2.3%–9% and 2.7%–12.1%, respectively (13,15,16,18–21,23–26,29,30). The low PCCRC frequency and the higher PCCRC rate for early lesions compared with that for deeply invasive lesions could be a consequence of their early detection by the high-quality colonoscopies performed; all achieved cecal intubation and were performed by endoscopy experts, and 62% of patients had histories of multiple negative colonoscopies. According to the policy that diminutive adenomatous polyps ≤5 mm without characteristics suggestive of cancer can be followed without resection (37–39), surveillance colonoscopies are performed at short intervals in clinical practice (40), which could contribute to PCCRC detection.

In this study, the PCCRCs were more likely to be detected in men, which differs from previous reports (14,18–21). Unlike the Tis and T1 lesions, the T2 or more deeply invasive cancers were more likely to be located in the proximal colon, which concurs with previous reports (13,14,16–18,20–29). Many Tis and T1 cancers appeared superficial, their coloring was similar to that of the surrounding normal mucosa, and they were detected soon after negative colonoscopies. Therefore, it is highly likely that the early-stage PCCRCs were missed lesions irrespective of their locations and were detected through high-quality colonoscopies. However, less superficial lesions, regardless of their T stage, were detected frequently in the sigmoid colon where folds and bends cause blind spots. Therefore, colonoscopists must take care not to miss less superficial lesions, especially in the sigmoid colon. Clearly, inadequate colonoscopies and poor endoscopic skills affect the frequencies at which lesions are missed (13–15,19,21,22,24). Furthermore, in this study, poor bowel preparation was observed in 11% of the previous negative colonoscopies in patients with Tis cancers.

Regarding the biological features of PCCRCs, 40% of T1 or more deeply invasive cancers exhibited dMMRs, which aligns with previously reported rates of 24%–32% (23,26,28–30); these lesions were more likely to be located in the proximal colon. Interestingly, only 6% of the Tis cancers exhibited dMMRs, which was a considerably lower rate than the rates for T1 or more deeply invasive cancers. This indicates that most of the Tis PCCRCs were slow-growing lesions, missed during previous colonoscopies. By contrast, 4 of 8 T2 or more deeply invasive cancers located in the proximal colon exhibited dMMRs; these may have been rapidly advancing cancers. Two of these lesions were mucinous or poorly differentiated adenocarcinomas, had *BRAF*^*V600E*^ mutations (suggesting carcinogenesis through the serrated pathway) (34), and were associated with poor prognoses; mutation analyses failed for the other 2 T2 or more deeply invasive cancers. Shaukat et al. (30) also reported that PCCRCs with *BRAF* mutations were more likely to be located in the proximal colon, be mucinous or poorly differentiated adenocarcinomas, have a high microsatellite instability, and be associated with poor prognoses. Thus, PCCRCs that progress through the serrated pathway grow rapidly and are associated with poor prognoses. Sessile serrated lesions, which are precursors of CRCs, also appear superficial, and their coloring is similar to that of the surrounding normal mucosa, and are easily missed, as described previously. Hence, superficial lesions require rigorous attention.

To date, studies have investigated a few gene mutations, e.g., *KRAS* and *BRAF*. In this study, the genomic mutations were analyzed exhaustively, and some characteristic genomic alterations were detected. We confirmed that PCCRCs with dMMRs were associated with hypermutations. One T3 PCCRC with pMMRs, detected <24 months after a negative colonoscopy (case 9), had an *MYC* missense mutation, which is inactivated by Wnt activation and the inactivation of transforming growth factor (TGF)-β signaling, and is strongly associated with CRC proliferation; therefore, it might be associated with tumor progression (41). *PIK3CA* encodes p110α, which is a catalytic subunit of PI3K that is associated with the proliferation of CRCs. *PIK3CA* mutated in 5 of 6 T2 or more deeply invasive PCCRCs, which was more frequent than that reported for CRC (27%) by The Cancer Genome Atlas (42). In addition, 4 of 5 tumors with *PIK3CA* mutations were located in the proximal colon, and *PIK3CA* mutations were not detected in the Tis/T1 PCCRCs; therefore, invasive PCCRCs in the proximal colon were strongly associated with *PIK3CA* mutations. *KRAS* and *BRAF* mutations were found in 52% (12/23) and 13% (3/23) of the tumors, respectively. Previous studies have shown that *KRAS* and *BRAF* mutations were present in 23%–29% (9,23,27,29) and 17%–28% (9,23,27,29,30) of PCCRCs, respectively; these rates did not differ from those in detected CRCs (9,27,29,30). The frequency of the *KRAS* mutation was higher in this study than in other studies, which may be due to fewer patients.

This study had some limitations. First, this was a single-center retrospective study with a relatively small number of patients. Although a large multicenter study could generate superior epidemiological results, it may generate less accurate information about the colonoscopies and lower quality colonoscopies. In this study, we could determine whether PCCRCs are related to missed lesions by investigating detailed and accurate data. Second, the genomic analyses were performed on a limited 23 of 34 PCCRCs because most others had insufficient quality and/or quantity DNA. Tumor sample storage was not consistent, and many tumors had been resected >5 years before they were analyzed. Third, the analysis of the patients with CRC during this study was based on the policy that diminutive polyps may be followed without resection. Therefore, PCCRCs in this study included many lesions that developed from previously detected diminutive polyps, which ought to be excluded from the analysis. Fourth, the number of PCCRCs in this study may have been underestimated compared with those in other population-based studies because we could not account for patients who had undergone previous colonoscopies in other hospitals. Finally, the adenoma detection rate of each colonoscopist, which can have a great impact on PCCRC (12), could not be evaluated. Although a large-scale prospective study with uniform colonoscopy intervals and high-quality colonoscopy is required, our study investigated the clinicopathological features, dMMR immunohistochemistry, genomic mutations of PCCRCs, and the interrelationship among these characteristics, and its findings indicated that PCCRCs are associated with missed lesions and rapidly growing cancers.

In conclusion, PCCRCs can be invasive cancers with dMMRs and/or *PIK3CA* mutations, which can grow rapidly, are associated with poor prognoses, and are located in the proximal colon, or missed early lesions that are either superficial lesions that can be located anywhere in the colon and rectum or less superficial lesions in the sigmoid colon. To prevent PCCRC, superficial lesions should not be missed during colonoscopy. Although these findings must be validated in prospective large-scale clinical trials, we believe that our findings inform the development of new CRC surveillance methods and the identification of a group of CRCs with a poor prognosis.

## CONFLICTS OF INTEREST

**Guarantor of the article:** Shiro Oka, MD, PhD.

**Specific author contributions:** Hidenori Tanaka, MD, and Yuji Urabe, MD, PhD, contributed equally to this work. All authors contributed to the study conception and design. H.T., Y.U., Y.S., T.N., K.I., Y.O., K.M., K.Y., K.S., Y.N., and R.Y.: collected and/or analyzed the data. H.T.: wrote the initial draft of the manuscript, Y.U. and S.O.: assisted in revising the manuscript, and all authors commented on previous versions of the manuscript. S.T. and K.C.: critically reviewed the manuscript, and K.C.: approved the final version of the manuscript. All authors approved the manuscript and agree to its submission for publication.

**Financial support:** This work was supported by the Program of the Network-type Joint Usage/Research Center for Radiation Disaster Medical Science. This study was supported by grants from the Japan Society for the Promotion of Science (JSPS KAKENHI Grant Number: 19K17493; http://www.jsps.go.jp/j-grantsinaid/), the Japanese Foundation for Research and Promotion of Endoscopy, and a Hiroshima University NOZOMI H Fund Grant for the Promotion of Cancer Research.

**Potential competing interests:** None to report.

**Study Highlights**

**WHAT IS KNOWN**

✓ PCCRCs may represent missed lesions.
✓ PCCRCs categorized as intramucosal lesions have never been investigated.
✓ The detailed genomic characteristics of PCCRCs have never been investigated.

**WHAT IS NEW HERE**

✓ Most early lesions are missed superficial lesions.
✓ PCCRCs with deficient MMRs and/or *PIK3CA* mutations are detected in the proximal colon.

## Supplementary Material

SUPPLEMENTARY MATERIAL
