# Supplementary figures and images for: Clinical Features and Genomic Characterization of Post-Colonoscopy Colorectal Cancer
Source: Clin Transl Gastroenterol. 2020 Oct 6;11(10):e00246. doi: 10.14309/ctg.0000000000000246 (PMC7544176; doi:10.14309/ctg.0000000000000246)

## Slide 1
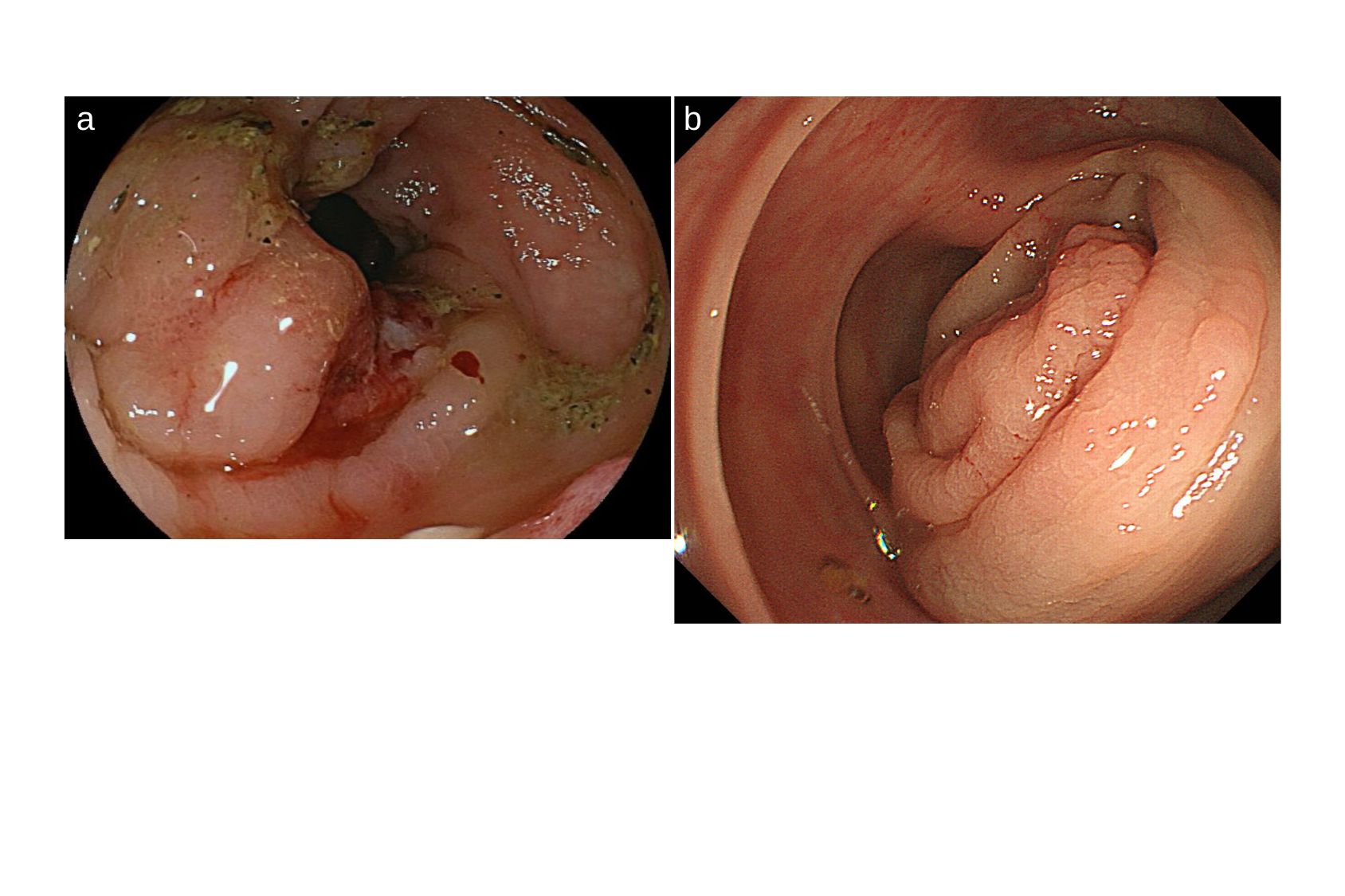

a
b

Supplement: SUPPLEMENTARY MATERIAL [file ct9-11-e00246-s004.pptx]
